# Malaria prevention in the city of Yaoundé: knowledge and practices of urban dwellers

**DOI:** 10.1186/s12936-019-2799-6

**Published:** 2019-05-09

**Authors:** Abdou Talipouo, Carmene S. Ngadjeu, Patricia Doumbe-Belisse, Landre Djamouko-Djonkam, Nadege Sonhafouo-Chiana, Edmond Kopya, Roland Bamou, Parfait Awono-Ambene, Sylvain Woromogo, Sevilor Kekeunou, Charles S. Wondji, Christophe Antonio-Nkondjio

**Affiliations:** 1 Institut de Recherche de Yaoundé (IRY), Organisation de Coordination pour la Lutte contre les Endémies en Afrique Centrale (OCEAC), P.O. Box 288, Yaoundé, Cameroon; 2 Faculty of Sciences, University of Yaoundé 1, P.O. Box 337, Yaoundé, Cameroon; 3 Faculty of Health Sciences, University of Buea, P.O. Box 456, Buea, Cameroon; 4 Faculty of Sciences, University of Dschang, P.O. Box 337, Dschang, Cameroon; 5 Centre Inter Etats d’Enseignement Supérieur en Santé Publique d’Afrique Centrale (CIESPAC), P.O. Box 1536, Brazzaville, Congo; 6 Vector Biology Liverpool School of Tropical Medicine Pembroke Place, Liverpool, L3 5QA UK

**Keywords:** Malaria, Knowledge, Practices, Households, ITNs, *Anopheles*, Yaoundé

## Abstract

**Background:**

Malaria prevention in Cameroon mainly relies on the use of ITNs. Although several free distribution campaigns of treated nets have been conducted across the country, bed net usage remains very low. A household survey was conducted to assess knowledge of the population and practices affecting treated net usage in the city of Yaoundé.

**Methods:**

A community-based descriptive cross-sectional survey was conducted in January 2017 in 32 districts of the city of Yaoundé. Parents (household head, spouse or an elder representative) who consented to the study, were interviewed using a structured pre-tested questionnaire. Interviews were conducted in French or English. A questionnaire consisting of 22 questions was administered to know (i) people’s knowledge and attitude on preventive measures; and, (ii) attitudes concerning the treatment of malaria and estimated amount spent for malaria prevention and treatment.

**Results:**

A total of 1643 household heads were interviewed. Over 94% of people interviewed associated malaria transmission to mosquito bites. The main methods used against mosquito bites were: treated bed nets (94%; n = 1526) and insecticide spray or coils (32.2%; n = 523). The majority of people interviewed reported using bed nets mainly to prevent from mosquito bites (84.4%, n = 1257), rather than for malaria prevention (47.3%). Knowledge and attitude analysis revealed that people with university or secondary level of education have better knowledge of malaria, prevention and treatment measures compared to those with the primary level (OR = 7.03; P < 0.001). Also, wealthy households were more aware of good practices concerning malaria prevention and treatment compared to poor ones. In the majority of districts of Yaoundé, over 50% of people interviewed per district, had good knowledge of malaria and prevention measures but less than 50% applied good practices concerning malaria treatment and prevention. The amount spent annually by a household for vector control was CFAF 11,589 ± 1133 (US$21.87 ± 2.14) and CFAF 66,403 ± 4012 (US$125.29 ± 7.57) for malaria treatment.

**Conclusion:**

The study indicated that, despite good knowledge of malaria and prevention measures, few people apply good practices. More sensitization needs to be done to improve adherence to good practices concerning malaria prevention and treatment.

**Electronic supplementary material:**

The online version of this article (10.1186/s12936-019-2799-6) contains supplementary material, which is available to authorized users.

## Background

The rapid unplanned urbanization affecting major sub-Saharan Africa cities is considered to be responsible for the proliferation of mosquitoes, such as *Anopheles* and *Culex* species, in the urban environment [1]. These insects are an important source of nuisance for populations and vectors of diseases such as malaria, filariasis and arboviruses. Among these diseases, malaria constitutes a major public health threat [2]. In Cameroon, the disease represents 30% of outpatient consultations, 24% of morbidity cases and 18.7% of mortality cases in healthcare units [3]. Because there is no available vaccine against malaria, vector control is the main prevention approach [particularly insecticide-treated nets (ITNs)]. Several studies have shown their significant impact in reducing malaria morbidity and mortality in endemic zones [4–6].

When over 60% of the community is covered, ITNs could have a community effect by providing protection to both users and non-users of treated nets [7]. According to the World Health Organization, of the 663 million clinical cases averted between 2001 and 2014, it is estimated that 69% were averted due to ITNs [8]. Bed nets have a double action: they are a physical barrier preventing human from mosquito bites and when they are impregnated they could confer a chemical barrier by killing or repelling mosquitoes coming into contact with the insecticide present in the net fibres. ITN efficacy depends on their physical integrity, the insecticidal effect on local mosquito species and the proportion of people using a net, among those with access [9, 10]. Because treated nets could only be effective if people acquire and use them regularly, having the correct knowledge of, attitudes towards and practices relevant to malaria control interventions is key. It is therefore important to determine the level of bed net usage by the population. Four key indicators have been proposed by Roll-Back Malaria for monitoring and evaluating treated nets usage on the field, these include: (i) the proportion of households that own at least a net; (ii) the proportion of households that own at least one ITN for 2 people; (iii) the proportion of the population with access to an ITN within the household; and, (iv) the proportion of the population that used an ITN the previous night [11]. The data generated can be used to improve management strategies for instance determine periods when to redistribute nets, the frequency at which nets are to be distributed or strengthen existing control programmes by including additional measures to achieve a sustainable control of the disease. In Cameroon the arsenal for malaria prevention includes the promotion of ITN use, intermittent preventive treatment for pregnant women and seasonal malaria chemoprevention for children aged 3–59 months in the northern part of the country [3]. Re-analyzing the ownership and usage rate of nets in Cameroon from the Demographic and Health Survey (DHS) of 2011 [12] indicated that the proportion of households owning at least an ITN was 36.4%, the proportion of the population that used an ITN the previous night was 7.6% and ratio of use to access was 0.71. It is considered nowadays that up to 77% of households own at least a treated net whereas the proportion of people using a net, among those with access, is estimated at 58% [13].

Household surveys conducted in different parts of the country identified several factors hindering the use of bed nets, such as feeling hot when sleeping under mosquito nets, sleeping under damaged nets, sleeping outdoor, not using nets regularly [14–19]. Following these limits, sensitization campaigns on television and radio were initiated by the Government. Although a study conducted few months after the first sensitization campaigns indicated an increase in ITN usage in the cities of Yaoundé and Douala [20], it is not known if these actions increased the usage of nets by the population. Moreover, it is not known whether or how socio-demographic factors, such as the level of education, the economic status, gender or age affects the use of treated nets by the population. The present study was conducted to assess the knowledge and practices of Yaoundé inhabitants concerning malaria prevention and treatment before the implementation of a larval control trial in this city.

## Methods

### Study sites

The study was conducted in Yaoundé, the capital city of Cameroon (3°51′ N 11°29′ E) and the second largest city of Cameroon, with about 3 million inhabitants [21]. The city is located within the Congo-Guinean phytogeographic zone characterized by a typical equatorial climate with two rainy seasons extending from March to June and from September to November. Yaoundé is situated 800 m above sea level and surrounded by many hills [14]. In Yaoundé, malaria transmission is considered holo-endemic and seasonal, with *Anopheles gambiae* sensu lato as the main vector [22, 23]. Average annual prevalence of *Plasmodium falciparum* in the general population is estimated to vary between 34 and 50% from the city centre to the periphery [24]. Children between 0 and 15 years old are considered to be the most affected. This age group comprised 75% of asexual parasite carriers, 85% of carriers of high parasitaemia and 83% of gametocyte carriers [24, 25].

**Fig. 1:**
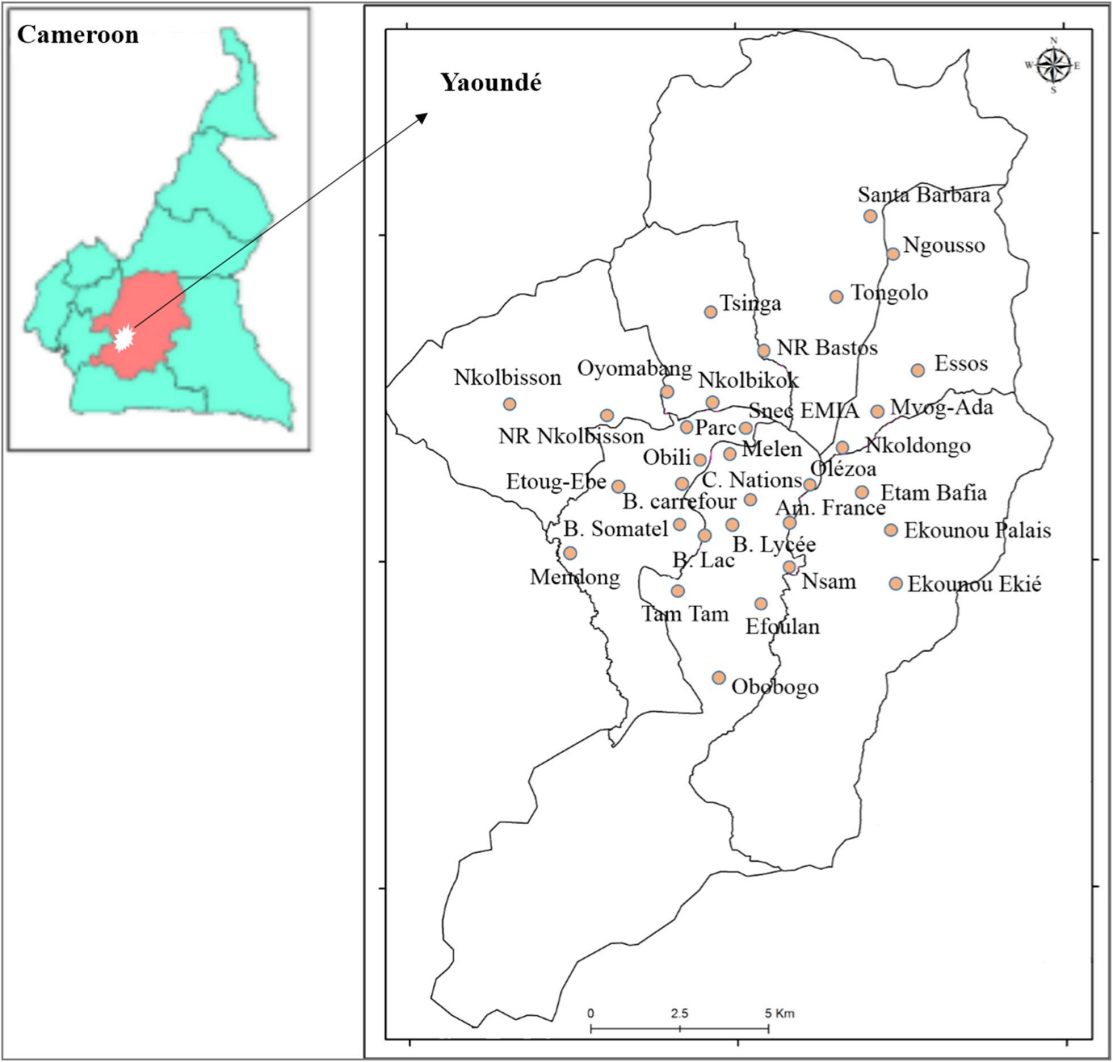
Map of Yaoundé showing studied districts

Investigations took place in 32 districts of the city of Yaoundé (Fig. 1). Selected districts were distributed from the periphery to the city centre and included highly populated, well-urbanized and spontaneously urbanized districts.

### Study design

The study was a community-based descriptive cross-sectional survey to assess population knowledge and attitude about malaria prevention and treatment in Yaoundé. A pre-tested questionnaire to assess the population basic knowledge on malaria, its vector and prevention measures was used for data collection (see Additional file 1). After preparing the questionnaire, internal reviews were undertaken by three researchers to assess the clarity of questions and their interpretability. A pilot study was subsequently conducted on a pool of 50 participants to test for validity, internal consistency and reliability of the questionnaire. The questionnaire was administered twice at different periods (after 1 week) to participants and the number of good answers provided to the different questions was scored to measure test–retest reliability. Participants were not informed on the second administration of the questionnaire.

Before the beginning of the survey, interviewers were trained on how to use the questionnaire and on methods to approach respondents and obtain consent. Interviewers were students or researchers with at least the master level. The survey was conducted in January 2017 during the long dry season. Parents (household head, spouse or and elder representative of the house) who consented to the study were interviewed. Interviews were undertaken in French or English and in private to reduce influence from other people. A questionnaire consisting of 22 questions was used to assess: (i) people’s knowledge and attitude on preventive measures; (ii) ownership and usage of ITNs; and, (iii) prevention measures. Most of the questions on knowledge and practices were drawn from the Malaria Indicator Survey (MIS) conducted in 2016 [26]. A certain number of demographic variables including the age, gender, level of education, and profession of the respondent, household composition and house construction materials were recorded as well. Only households where consent forms were approved were included in the study.

### Data analysis

Data recorded were registered into Microsoft Excel database. Data cleaning was performed to check for inconsistencies in data entry and responses. Data were analysed using SPSS version 20 statistical software package. Means, frequencies and proportions were used for descriptive analysis of the data. Percentages were compared using Chi squared test. Comparison between means was assessed using ANOVA. Different outcomes were also evaluated (i) the proportion of households that own at least a net; (ii) the proportion of households that own at least one ITN for 2 people; (iii) the proportion of the population with access to an ITN within the household; and, (iv) the proportion of the population that used an ITN the previous night. To identify factors associated with knowledge on malaria and usage of protection measures, the odds ratios (OR) as well as their 95% confidence intervals (95% CI) was computed using MedCalc v14.8.1 software. Statistical significance was set at P < 0.05. A multivariate logistic regression analysis was conducted to identify factors associated with the variable of interest. All variables significantly associated with the dependent variable in univariate analysis and variables with P value ≤ 0.15 were introduced in the model. Based on the type of houses, source of water used, and head of family job, a socio-economic indicator was created to classify households into poor and not poor. Houses constructed with mud, cemented walls or plank, using water from well and where parents have as occupation small business (not earning enough money to cover the household needs) were considered as poor. Houses constructed with brick and cement well equipped, using tap water and where parents had good jobs (earning enough money to cover the household needs) were considered as not poor or wealthy. To assess the knowledge of respondents on malaria, the answers to four different questions including malaria signs and symptoms, mode of transmission, measures of prevention and knowledge of mosquito breeding habitats were combined. Participants providing correct answers to at least three of the questions were considered as having a good knowledge of malaria. Those who had fewer than three correct answers were considered as having poor knowledge of malaria. Concerning good practices in regard to malaria prevention and treatment, the answers to four different questions including sleeping under a treated bed nets regularly, going to hospital for malaria treatment, eliminating standing water bodies around houses and purchasing drugs in the pharmacy were assessed. Participants providing appropriate answers to at least three of the questions were considered as applying good practices while those with fewer than three correct answers were considered having poor practices.

## Results

### Socio-demographics characteristics of participants

A total of 1643 households were surveyed during the study with a minimum of 50 households interviewed per district. Out of the 1643 households heads interviewed, 64.3% (N = 1031) were females and 35.7% (N = 572) males. The age range of people interviewed varied from 17 to 55 years old. The highest level of education attended by the majority of respondents (58.5%) was secondary school level, 18.6% had the primary level and 22.9% had the university level (Table 1). The majority of families heads interviewed reported doing small-scale business (60.2%). Houses were mainly constructed with cement blocks and tap water was commonly available in households.

**Table 1:** Socio-demographic characteristics of households surveyed in Yaoundé in January 2017

Items	Characteristics	N	Frequency (%)
Gender	Male	572	35.7
	Female	1031	64.3
Number of people in households	1–5	904	55.2
	6–10	612	37.4
	> 10	121	7.4
Highest level of education completed	Primary level	213	18.6
	Secondary level	671	58.5
	University level	262	22.9
Occupation	Public servant	358	23.6
	Small scale business	915	60.2
	Housewife	165	10.9
	Student	81	5.3
Type of constructions	Cements blocks	1095	68.4
	Mud and cement	165	10.3
	Clay	104	6.5
	Plank	237	14.8
Where do you fetch water?	Tap water	1139	71.4
	Well	262	16.4
	Natural source	71	4.5
	Drilling water	123	7.7

### General knowledge on malaria

People’s knowledge of vectors, use of protection measures, mosquito breeding habitats, symptoms of malaria, are presented in Table 2. The majority of respondents (94.9%, N = 1415) attributed the cause of malaria to mosquito bite. A high number of participants reported using treated bed nets (94%, N = 1526) for malaria prevention. Other measures used included insecticide spray or coils (32.2%, N = 523) and windows screens (5%, N = 82). Ranking their choices concerning why they were using treated nets, the majority of participants (84.4%) responded that they were using treated nets as a means of protection against mosquito nuisance (bites) as first or second choice, while only 47.3% responded that they were using bed net to prevent malaria transmission as first or second choices. Out of the 224 people reporting not using bed nets, 49% of the respondents (N = 110) indicated they did not use nets because they felt hot when sleeping under the net, some said it was because they did not possess a bed net (38.4%, N = 86), or that they had omitted hanging the net (6.7%, N = 15) and some reported the sensation of suffocating when sleeping under a net (5.8%, N = 13). The majority of bed nets available in households surveyed were freely acquired (94.8%, N = 1453) from the national free distribution campaigns and the remainder were either bought on the market or were gifts from relatives.

**Table 2:** Population knowledge, and behavior concerning the mode of transmission, use of preventive methods of malaria and larval habitats management

Variables	Answers	N	Frequency (%)
Mode of transmission of malaria	Mosquito bites	1445	94.9
	Dirt	54	3.5
	Cold	4	0.3
	Do not know	19	1.3
Preventive measures	Using mosquito nets	1526	94.0
	Using insecticides spray/coil	523	32.2
	Using net on windows	82	5.0
Role of mosquito nets	Preventing mosquito bites	1257	84.4
	Preventing malaria	705	47.3
	Preventing mosquito sing	43	2.9
	Sleeping well	62	4.2
Reasons for non-use of bed nets	Heat	110	50.0
	Omission	15	6.8
	Suffocation	13	5.9
	Not possessing a bed net	86	39.0
Origin of bed nets used	Freely acquired	1453	94.8
	Bought	148	9.7
Age of bed nets used	< 6 months	666	43.5
	> 6 months	455	29.9
	> 1 year	118	7.7
	> 2 years	288	18.9
Mosquito breeding sites	Stagnant water	770	51.5
	Gutters	386	25.8
	Swamp	155	10.4
	Dirt	383	25.6
	Bushes	92	6.2
	Do not know	84	5.6
Management of mosquito breeding sites	Draining	648	49.6
	Cleaning	339	26.0
	Treatment	34	2.6
	Do not know	302	23.1
Physical integrity of bed nets (N = 1523)	Damaged	629	41.4
	Undamaged	892	58.6

Percentages do not add up to 100 because these results are from multiple response questions

Concerning ITN ownership, the proportion of households owning at least a net varied from 82.3 to 100%. The proportion of households possessing an ITN for two people varied greatly according to districts from 42.2 to 76%. The proportion of the population with access to an ITN within their household varied from 41.1 to 57.7%. The proportion of the population that used an ITN the previous night varied from 65.7 to 95.5% (Table 3).

**Table 3:** Ownership and usage of insecticide-treated nets in households in districts of Yaoundé

Districts	% HHs owning ≥ 1 ITN	% HHs owning ≥ 1 ITN for 2 people	% population with access to an ITN within their own HH	% population that used an ITN the previous night
Ambassade de France	96.6	64.3	53.1	82.9
Biyem assi Carrefour	90.4	55.3	47.3	67.8
Biyem assi Lac	90.0	73.3	55.4	88.8
Biyem assi Lycée	92.0	71.7	53.9	85.1
Biyem assi Somatel	90.4	73.9	54.4	78.4
Cité des Nations	82.3	73.8	50.9	77.0
Efoulan Lac	100	64.0	51.7	81.6
Ekounou Ekie	92.3	58.3	46.7	75.8
Ekounou Palais	96.3	50.9	42.4	72.2
Essos	96.2	62.7	49.7	81.7
Etam Bafia	100	45.1	47.1	81.5
Etougebe	92.0	50.0	45.4	74.5
GP Melen	88.0	59.1	48.5	76.0
Mendong	92.1	44.7	44.4	69.1
Mvog Ada	92.1	62.2	51.8	75.7
Ngousso	94.2	49.0	44.8	72.2
Nkolbikok	91.8	42.2	41.1	65.7
Nkolbisson	92.0	43.5	40.3	66.8
NR Bastos	88.5	65.2	48.6	76.8
NR Nkolbisson	96.2	76.0	57.7	73.8
NR Nkoldongo	92.2	50.0	47.2	71.1
Nsam	90.2	68.8	50.9	77.1
Obobogo	88.8	46.8	47.7	77.4
Olezoa	100	68.0	49.8	74.2
Oyomabang	96.0	56.2	51.4	78.1
Parc Matgénie	98	67.3	49.6	95.5
Santa Barbara	94.2	42.8	42.7	73.1
Shell Obili	92.3	60.4	54.1	81.5
Snec EMIA	94.0	51.1	45.6	77.2
Tam Tam	88.0	61.4	50.2	84.8
Tongolo	100	51.9	49.6	78.3
Tsinga	92.4	66.6	51.8	72.8
Overall	99.7	58.5	66.2	76.7

*HH* household

People’s knowledge of mosquito breeding habitats and their management were assessed. The main mosquito breeding habitat mentioned by respondents were stagnant water bodies (51.5%, N = 770), followed by gutters (25.8%, N = 386), dirt (25.6%, N = 383), swampy areas (10.4%, N = 155), and bushes (6.2%, N = 92). About 5.6% (N = 84) of people say they did not know where mosquito breeds. Draining mosquito breeding sites (49.6%, N = 648), cleaning (26%, N = 339) or treatment of sites (2.6%, N = 34) were the most frequently mentioned management options for larval breeding habitats. Still, 23.2% (N = 302) of the respondents could not mention any management option of larval breeding sites.

Concerning malaria symptoms, people were asked to cite symptoms that they attribute to malaria. As first or second choice, over 80% of respondents included fever in their answers, whereas over 40% included headache. Others symptoms mentioned were backache (18.5%), fatigue (17.5%), vomiting (10.83%), and anorexia (4%).

### Home management of malaria cases and financial cost of vector control and malaria treatment

Out of 1590 household heads interviewed, the majority (60.5%; N = 963) reported practicing self-medication when they suspect a case of malaria (Table 4). About 34.3% (N = 545) and 5.2% (N = 82) of respondents reported going to hospital or clinic for consultation and using traditional medicine, respectively. The majority of respondents (72.7%; N = 1078) who practiced self-medication reported buying drugs in pharmacy. Some of the participants reported buying their drugs to street-sellers (36.2%; N = 537) or in hospital (20.6%; N = 306) or using plants. In average, annual expenses of a household for vector control and malaria treatment was estimated at CFAF 11,589 ± 1133 (US $21.87 ± 2.14) and CFAF 66,403 ± 4012 (US $125.29 ± 7.57), respectively. Significant variation in the amount spent by households according to districts was recorded for both mosquito control (F = 7.951; P < 0.001) and malaria treatment (F = 1.549; P = 0.03).

**Table 4:** Home management of malaria cases in households in Yaoundé in January 2017

Items	Characteristics	N (frequency)
Management of malaria cases (N = 1590)	Hospital consultation	545 (34.3%)
	Traditional^a^	82 (5.2%)
	Self-medication	963 (60.5%)
Buying drugs (N = 1482)	Pharmacy	1078 (72.7%)
	Street drugs	537 (36.2%)
	Traditional^a^	80 (5.4%)
	Hospital	306 (20.6%)
Expenses	For mosquito control	11,589 ± 1333
	For malaria treatment	66,403 ± 4012

Percentages concerning buying drugs do not add up to 100 because these results are from multiple response questions

^a^Traditional: use plants for malaria treatment

### Relationship between the level of education and knowledge of malaria and usage of protection methods

Comparisons were conducted to assess any association between good knowledge of malaria, good practices and education level. From the analysis, it appeared that participants who had the university or secondary levels had a better knowledge of malaria compared to those with the primary level (OR = 7.03; P < 0.001). Also, participants with the university or secondary levels of education were more aware of good practices concerning malaria prevention and treatment compared to those with the primary level (OR = 1.61; P = 0.03) (Table 5).

**Table 5:** Factors associated with good knowledge and practices about malaria

Factors	Categories	N	% with good knowledge	OR (95% CI)	P	N	% with good practices	OR (95% CI)	P
Level of education	Primary	188	87.8	1.0		186	40.3	1.0	
	Secondary	466	92.7	1.7 (1.0–3.1)	0.04	464	45.7	1.2 (0.9–1.7)	0.21
	University	206	98.5	7.0 (2.4–20.7)	< 0.001	205	48.8	1.4 (0.9–2.1)	0.09
Socio-economic status	Poor	1327	92.2	1		1316	44.7	1	
	Not poor	224	93.8	1.27 (0.7–2.3)	0.4	222	50.5	1.3 (0.9–1.7)	0.11
Gender	Male	569	88.6	1		562	48.9	1	
	Female	1028	91.2	1.4 (1–1.9)	0.08	1022	48.8	0.99 (0.8–1.2)	0.96
Occupation	Small business	641	89.7	1		640	45.6	1	
	Public servant	287	92.7	1.45 (0.8–2.4)	0.15	285	54.7	1.44 (1.1–1.9)	0.01
	Housewife	130	89.2	0.95 (0.5–1.7)	0.87	131	46.6	1.03 (0.7–1.5)	0.84
	Student	81	95.1	2.2 (0.8–6.2)	0.13	80	47.5	1.07 (0.7–1.7)	0.75
Having a window screen	No	1538	90.2	1		1526	49.0	1	
	Yes	82	97.6	4.3 (1.1–17.7)	0.04	82	42.7	0.8 (0.5–1.2)	0.26

### Relationship between socio-economic status and knowledge of malaria and usage of prevention methods

Comparisons were also conducted to assess potential associations between good knowledge of malaria, good practices and socioeconomic status of the household. From the analysis, it appeared that households of good economic status were more aware of or applying good practices concerning malaria prevention and treatment compared to the poor ones (OR = 2.34; P < 0.001). However, no significant association was found between socioeconomic status and knowledge of malaria (OR = 1.27; P = 0.40) (Table 5).

To assess the level of association between practices and knowledge and some measured indicators, a multivariate analysis with good knowledge or practices as outcome variable and different measured parameters (gender, education level, occupation, economic status, presence of window screens) as explanatory variables was undertaken. When analyses were performed with best practices as outcome variable, the presence of screens on windows and university or secondary education level exhibited strong positive association with best practices (P < 0.05). When good knowledge was considered as outcome variable, the following explanatory variables were recorded significantly associated with good knowledge: gender (women), education level (secondary or university level) and economic status (wealthy) (P < 0.05).

### Spatial distribution of good knowledge and good practices in districts of Yaoundé

**Fig. 2:**
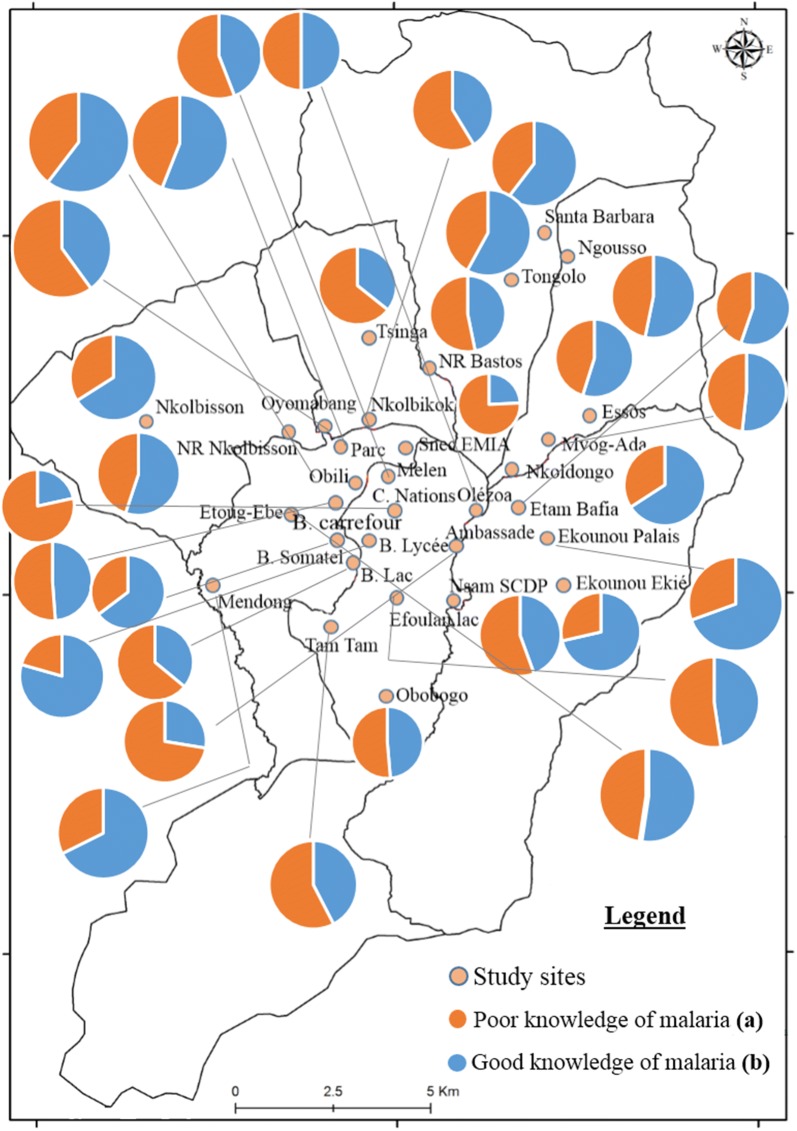
Spatial distribution of good and poor knowledge of the population concerning malaria prevention in the city of Yaoundé in January 2017. Good knowledge: Proportion of people who provided at least 3 correct answers out of the questions concerning: malaria signs and symptoms, mode of transmission of malaria, measures of prevention and knowledge of mosquito breeding habitats. Poor knowledge: Proportion of people not able to provide at least 3 correct answers to the 4 questions mentioned above)

**Fig. 3:**
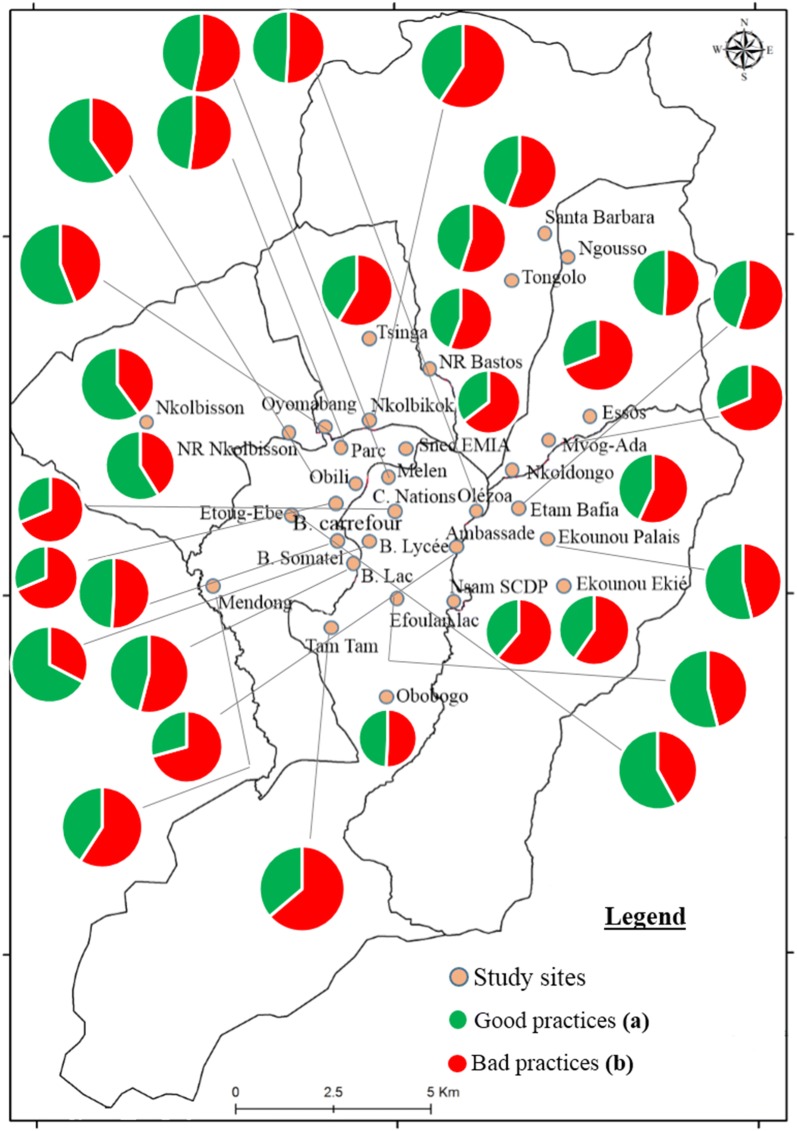
Spatial distribution of good and bad practices of the population concerning malaria in the city of Yaoundé in January 2017. Good practices: Proportion of people who provided 3 good answers out of 4 from the following points: sleeping regularly under a treated net, going to the hospital for malaria treatment, eliminating standing water bodies around houses and purchasing drugs in the pharmacy. Bad practices: Proportion of people having less than 3 good answers

Significant variations were recorded when comparing knowledge and practices between districts (P ˂ 0.002). From the analysis, it appeared that in most districts, more than 50% of people interviewed had good knowledge of malaria and prevention measures (Fig. 2). Concerning practices, it appeared that in 24 out of 32 districts less than 50% of people interviewed apply good practices concerning malaria treatment and prevention (Fig. 3).

## Discussion

The study main objective was to assess the level of awareness and attitude of Yaoundé population on malaria prevention. A high proportion of people interviewed had good knowledge of malaria, its vector and methods of protection. These findings were in line with previous reports conducted in the country [14, 15, 17–19]. Yet, the level of knowledge was found to vary according to the education level and socio-economic status of respondents. Thus, well-educated people (university level) had good knowledge of malaria prevention measures and of the treatment compared to those with primary level. The following was consistent with studies conducted elsewhere [27, 28]. This could be explained by the fact that malaria is taught in school, and because educated people are more likely to be reached by malaria messages on different audio-visual platforms such as television, radio, newspapers, internet, while this is not the case for less educated people. In order to increase knowledge and awareness of the community about malaria and its prevention, additional sensitization tools need to be used such as: community educators, focus group discussion or social media (Facebook, WhatsApp, YouTube), which are now widely used by the population. Yet, the use of social media at the national level to inform the population is still not widespread and could be a good means for communicating with the population [29]. Wealthier people were more likely to apply good practices concerning malaria prevention and treatment compared to the poor ones. However, no significant difference in knowledge was recorded between households classified as poor and not poor and could come from the limited sensitivity of indicators used for discriminating the two groups.

The proportion of households possessing at least a net was high. This number decreases significantly when the proportion of households having one bed net for two people was considered despite frequent mass distributions of bed nets to the population in the country [3]. The following stresses the need for the use of good indicators to assess ownership and usage of treated nets. The majority of bed nets found in households came from the last free-of-charge distribution campaign conducted in 2015 by the National Malaria Control Programme (NMCP). However, some households reported preferring using ITNs distributed in 2011, because they were larger than those distributed in 2015. The following demonstrates the need for more practical considerations when choosing nets for the population. Further, over 41% of people interviewed said they had nets partly or completely damaged. This rapid degradation of mosquito net 2 years after distribution could result from the frequent or bad utilization of this tool by the population or to the poor quality of material used. Treated nets distributed to the population during mass distribution campaigns included the following brands: Interceptor^®^, Olyset^®^, Duranet^®^, and Permanet 2.0^®^ [26]; all these nets brands are approved by the WHO for malaria prevention. Studies conducted in Burundi and Kenya also revealed a high rate of physical deterioration of nets after only a year of use [30, 31]. This highlights the need for regular follow-up of net durability or physical integrity to determine likely periods for bed net redistribution campaigns to maximize the impact of treated nets. The WHO recommends that because ITNs could be effective for at least 3 years under field conditions, that mass distribution campaigns should be conducted every 3 years [32–34]. Moreover, it is stated that strategies to target at-risk groups through continuous bed net distribution should be considered [35]. Although treated nets provide both a chemical and a physical barrier to mosquito bites by reducing the contact between mosquito and man, when nets are highly damaged they become less efficient against mosquitoes. Fabrics commonly used to produce bed nets include polyester, polyethylene, polypropylene, cotton, cotton-synthetic blends and nylon. Polyester is the most popularly used fabric, because it is lighter and allows more air movement whereas cotton nets although more solid are less comfortable. To avoid mosquito biting through the net it is important to use sufficiently large nets. Rectangular nets provide enough room for someone to sleep in without being bitten by mosquitoes. This proves the need for a good choice of the quality and size of nets to be distributed to the population in mass distribution campaigns.

It is possible that the true proportion of residents who slept under an ITN the previous night could be much lower than estimated since self-report was used to measure net use in households. This could have overestimated the actual usage of ITNs since in most districts, > 70% of people reported having used a net the previous night. These values are 10 to 30% higher than the average estimates at the national level estimated at 58% [26]. Self-reported measures have been found to overestimate ITN adherence by over 13% elsewhere [36].

Several factors were recorded hindering the use of bed nets in the population, including feeling hot when sleeping under a net, not using nets regularly, not possessing a net. Up to 38% of people not using nets reported not possessing a net. Similar observations were reported in previous surveys [14]. These factors highlight the need for additional measures in order to improve bed net ownership and utilization. In Zambia, door-to-door delivery of ITNs to households in remote areas associated with net hanging and face-to-face health education on ITN use and ways of reducing net wear and tear were practiced and allowed higher coverage rates [37]. Drafting of key messages to disseminate information and their appropriate delivery through interpersonal communication, mass and print media coupled with hands-on instructions to householders on net hanging and maintenance was also found to increase community awareness and uptake of malaria interventions [38]. Furthermore, because the usage of treated nets varied according to districts, mapping the urban domain according to the usage rate of treated nets could be helpful for targeting districts needing further sensitization. Interventions in schools could also be an interesting option for sensitization since they are well distributed geographically and thus provide access to a large proportion of the targeted group. Moreover, children are considered as changed agents and targeting them can potentially lead to improved ITN use within the household [37, 39].

In several households, the use of ITNs was associated with other means of prevention against mosquito bites such as: insecticide spray, coils, repellents, or the use of screens on windows. This was particularly the case for people living near marshland where the nuisance due to mosquitoes could be very important. The use of insecticide spray and coils alongside pesticides in agriculture are considered to increase insecticide selective pressure in mosquito populations and to induce insecticide resistance in mosquito populations [23, 40–42]. Although ITNs are considered a first-line intervention tool, this tool is not 100% effective against resistant mosquito populations or outdoor-biting mosquitoes and need to be complemented with other control tools, such as larval control when there are indications of rapid evolution of insecticide resistance. This could justify the need to implement a larviciding programme in a city such as Yaoundé to complement existing interventions. Larval control has been reported to reduce malaria transmission in urban setting [43, 44] and could be an important component for the control and elimination of malaria in the city of Yaoundé [45].

About 60% of respondents reported doing self-medication and 34.3% reported going to hospital for consultation when they suspect a case of fever. A high proportion of people doing self-medication was recorded among well-educated people. The recourse to self-medication could be explained by the fact that most people think they will spend a lot of money and much of their time if they go to the hospital. These findings are similar to reports from previous studies [46, 47]. As the study demonstrated, taking in charge a case of malaria could be very costly for poor households. To address these issues the Government of Cameroon is subsidizing the treatment of malaria cases in healthcare facilities and since 2010 several community health workers have been trained to provide first care to people suffering from uncomplicated malaria or common diseases in the population [13]. Because people are not always aware of the existence of these practitioners in the community, more information and sensitization need to be done towards this end. In addition to that, it came out from the study that more and more people purchase drugs for malaria treatment from street sellers. This situation is of paramount importance and need to be dealt with as urgently as possible. Because drugs that are sold on the streets are not maintained in good condition and are of poor quality, they could be the source for rapid spread of drug resistance. Special measures need to be undertaken to control the selling and consumption of these drugs.

## Conclusion

The study revealed a heterogeneous pattern concerning knowledge and usage of prevention measures by the population and stresses the need for implementing additional sensitization approaches, such as community educators, focus group discussion or social media (Facebook, WhatsApp, YouTube) to reach more people. Moreover, because malaria vectors have been reported to have become increasingly resistant to insecticide in the city of Yaoundé, the use of an integrated control approach with larviciding coming as a complement to existing control tool could be indicated. In this context, assessing population adherence to this new control intervention could be important to determine the sustainability of such an approach in the control of malaria in the city of Yaoundé.

## Additional file

**Additional file 1.** Questionnaire on population knowledge and attitude on malaria.

## Data Availability

Not applicable.

## Abbreviations

ITNs: insecticide-treated nets
RBM: Roll Back Malaria
IPTP: intermittent preventive treatment for pregnant women
NMCP: National Malaria Control Programme
