# Correction to: Malaria prevention in the city of Yaoundé: knowledge and practices of urban dwellers

**DOI:** 10.1186/s12936-019-2839-2

**Published:** 2019-06-20

**Authors:** Abdou Talipouo, Carmene S. Ngadjeu, Patricia Doumbe-Belisse, Landre Djamouko-Djonkam, Nadege Sonhafouo-Chiana, Edmond Kopya, Roland Bamou, Parfait Awono-Ambene, Sylvain Woromogo, Sevilor Kekeunou, Charles S. Wondji, Christophe Antonio-Nkondjio

**Affiliations:** 1 Institut de Recherche de Yaoundé (IRY), Organisation de Coordination pour la Lutte contre les Endémies en Afrique Centrale (OCEAC), P.O. Box 288, Yaoundé, Cameroon; 2 Faculty of Sciences, University of Yaoundé 1, P.O. Box 337, Yaoundé, Cameroon; 3 Faculty of Health Sciences, University of Buea, P.O. Box 456, Buea, Cameroon; 4 Faculty of Sciences, University of Dschang, P.O. Box 337, Dschang, Cameroon; 5 Centre Inter Etats d’Enseignement Supérieur en Santé Publique d’Afrique Centrale (CIESPAC), P.O. Box 1536, Brazzaville, Congo; 6 Vector Biology Liverpool School of Tropical Medicine Pembroke Place, Liverpool, L3 5QA UK

## Correction to: Malar J (2019) 18:167 10.1186/s12936-019-2799-6

Following publication of the original article [1], the authors flagged an error concerning Table 3.

In Table 3, the values in the fourth column (% population with access to an ITN within their own HH) and some values in the fifth column (% population that used an ITN the previous night) are incorrect. The corrected Table 3 is given here (all changes in the table are italicized).

**Table 3:** Ownership and usage of insecticide-treated nets in households in districts of Yaoundé

Districts	% HHs owning ≥ 1 ITN	% HHs owning ≥ 1 ITN for 2 people	% population with access to an ITN within their own HH	% population that used an ITN the previous night
Ambassade de France	96.6	64.3	*85.7*	82.9
Biyem assi Carrefour	90.4	55.3	*76.3*	67.8
Biyem assi Lac	90.0	73.3	*88.3*	*87.8*
Biyem assi Lycée	92.0	71.7	*85.3*	*81.5*
Biyem assi Somatel	90.4	73.9	*88.7*	78.4
Cité des Nations	82.3	73.8	*81.9*	77.0
Efoulan Lac	100	64.0	*86.4*	81.6
Ekounou Ekie	92.3	58.3	*81.2*	75.8
Ekounou Palais	96.3	50.9	*72.6*	72.2
Essos	96.2	62.7	*82.4*	81.7
Etam Bafia	100	45.1	*83.2*	81.5
Etougebe	92.0	50.0	*76.4*	74.5
GP Melen	88.0	59.1	*82.1*	76.0
Mendong	92.1	44.7	*78.7*	69.1
Mvog Ada	92.1	62.2	*83.7*	75.7
Ngousso	94.2	49.0	*78.8*	72.2
Nkolbikok	91.8	42.2	*73.4*	65.7
Nkolbisson	92.0	43.5	*72.9*	66.8
NR Bastos	88.5	65.2	*84.2*	76.8
NR Nkolbisson	96.2	76.0	*90.3*	73.8
NR Nkoldongo	92.2	50.0	*79.2*	71.1
Nsam	90.2	68.8	*84.4*	77.1
Obobogo	88.8	46.8	*83.7*	77.4
Olezoa	100	68.0	*85.1*	74.2
Oyomabang	96.0	56.2	*80.2*	78.1
Parc Matgénie	98	67.3	*79.3*	*75.2*
Santa Barbara	94.2	42.8	*76.0*	73.1
Shell Obili	92.3	60.4	*83.7*	81.5
Snec EMIA	94.0	51.1	*82.5*	77.2
Tam Tam	88.0	61.4	*85.8*	84.8
Tongolo	100	51.9	*80.3*	78.3
Tsinga	92.4	66.6	*84.3*	72.8
Overall	99.7	58.5	*81.4*	*76.1*

HH: household

In addition, consequent of the errors in Table 3, please be advised of these corrections to the second paragraph under subsection ‘General knowledge on Malaria’ (with the corrections in italics):

The proportion of the population with access to an ITN within their household varied from *72.6 to 90.3%.* The proportion of the population that used an ITN the previous night varied from 65.7 to *87.8%* (Table 3).

The authors apologize for this error.

## References

[1] Talipouo A, Ngadjeu CS, Doumbe-Belisse P, Djamouko-Djonkam L, Sonhafouo-Chiana N, Kopya E, Bamou R, Awono-Ambene P, Woromogo S, Kekeunou S, Wondji CS (2019). Malaria prevention in the city of Yaoundé: knowledge and practices of urban dwellers. Malar J.

