# Supplementary material for: Malaria prevention in the city of Yaoundé: knowledge and practices of urban dwellers
Source: Malar J. 2019 May 9;18:167. doi: 10.1186/s12936-019-2799-6 (PMC6509831; doi:10.1186/s12936-019-2799-6)
Supplement: Supplementary file 1 — Additional file 1. Questionnaire on population knowledge and attitude on malaria. [file 12936_2019_2799_MOESM1_ESM.doc]

**
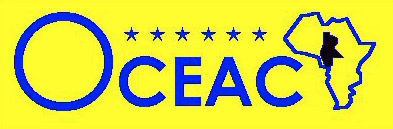
ORGANISATION DE COORDINATION POUR LA LUTTE CONTRE
LES ENDEMIES EN AFRIQUE CENTRALE**

Contacts :

-Dr N. Antonio : 699 53 86 56

- Dr A. Parfait : 699 836 111

**PO Box 15665, Yaoundé Cameroun**

**Phone : + 237 22 23 22 32**

**Fax : + 237 22 23 00 61**

**Web : http:// www.oceac.org**

***Questionnaire on population knowledge and attitude on malaria***

| **0. IDENTIFICATION** | | | | | | |
| --- | --- | --- | --- | --- | --- | --- |
| 0.1 | **Date** | | |___||___|/|___||___|/2017 | | | |
| 0.2 | **Interviewer** | |  | | | |___||___| |
| 0.3 | **District** | |  | | | |___||___| |
| 0.4 | **Cluster** | |  | | | |___||___| |
| 0.5 | **House N°** | |  | | | |___||___| |
| 0.6 | **GPS coordinates (WGS84)** | | 1. Latitude 2. Longitude | |___||___||___||___||___||___|  |___||___||___||___||___||___| | | |
| 0.7 | **Name of the head of household** | | *_________________________________________________* | | | |
| 0.8 | **Level of education of the head of household** | | 1 = University ; 2. = High school (form 6&7);  3 = Secondary level (form 1-5); 4 = Primary ;  5 = No level | | | |___| |
| 0.9 | **Main activity of the head of household (job)** | |  | | |  |
| 0.10 | **Name of the respondent (if different from the head of household)** | | _________________________________________________ | | | |
| 0.11 | **School level of the person interviewed** | | 1 = University ; 2. = High school (form 6&7);  3 = Secondary level (form 1-5); 4 = Primary ;  5 = No level | | | |___| |
| 0.12 | **Main source of water supply** | | 1 = Tap water ; 2 = Pipped outside house ; 3 = well ; 4 = Natural source | | | |___| |
| 0.13 | **Type of house** | | 1 = Cement/Brick; 2 = Mud and cement ; 3 = Plank ; 4 = Mud | | | |___| |
| **1. ATTITUDE TOWARDS MALARIA** | | | | | | |
| 1.1 | **How many people live in this house ?** | 1. Total number 2. Children of less than 5 years | | | |___||___|  |___||___| | |
| 1.2 | **According to you what is responsible for malaria transmission to human ?** | ___________________________________________________________________________ | | | |___||___| | |
| 1.3 | **What can you use to prevent from malaria?** | ___________________________________________________________________________ | | | |___| | |
| 1.4 | **What does people in this house do to stop mosquitoes from biting them?** | 1. Bednets ; 2. Screening windows and eaves ; 3. Mosquito repellents 4. Fan ; ***1 = Yes 2 = No*** 5. Coils 6. Insecticides spray 7. Air onditionner 8. Other (give precision) __________________ | | | |___|  |___|  |___|  |___|  |___|  |___|  |___|  |___| | |
| 1.5 | **If bed nets are mentioned when are bed nets mostly used?**  ***(If answer to 1.4.A = 1)*** | 1 = Rainy season ; 2. = Dry season ;  3 = Regularly  4= other (give precision) __________________ | | | |___| | |
| 1.6 | **Since how long do you have bed nets ?** | 1 = les than 6 months ; 2. = more than 6 months ;  3 = More than a year ; 4 =. More than 2 years | | | |___| | |
| 1.7 | **How did you obtain your bed nets ?** | 1 = Free distribution campaigns ; 2. = buy ;  3 = from a donor (differnt from MINSANTE) ;  4 = other precise_______________ | | | |___| | |
| 1.8 | **Does all beds in the house have treated nets ?** | 1= Yes 2= No | | | |___| | |
| 1.9 | **How many bed nets are in the house ?** | _____________________________________ | | | |___||___| | |
| 1.10 | **Who slept under a bed net last night ?** | 1. Everybody ; 2. Parents ; 3. All the children 4. Children of <5years ; 5. Visitors 6. Other (give precision) _________________ | | | |___|  |___|  |___|  |___|  |___|  |___| | |
| 1.11 | **Who regularly sleep under a bed net in the house?** | 1. Everybody ; 2. Parents ; 3. All the children 4. Children of <5years ; 5. Visitors 6. Other (give precision) _________________ | | | |___|  |___|  |___|  |___|  |___|  |___| | |
| 1.12 | **Why do you use mosquito nets ?** | 1 =For preventing mosquito bites  2 = To avoid mosquitoes singing  3 = To be able to sleep ;  4. = To prevent malaria attacks ;  5 = Other (give precision)__________________ | | | |___| | |
| 1.13 | **What is the main reason why people don’t use bed nets in this house?** | 1 = Forget ; 2 = Heat ;  3 = No mosquito in the house  5 = Other (give precision) ____________________ | | | |___| | |
| 1.14 | **In which conditions are your bed nets** ? | 1 = Not damaged (no holes)  2 = Damaged with many holes  **(Give the number)** | | | |___||___|  |___||___| | |
| 1.15 | **Do you know where mosquito larvae breed ?** | 1= Yes 2= No | | | |___| | |
| 1.16 | **Can you cite some breeding habitats for mosquitoes ?** | __________________________________________________________________________________________________________________ | | | |___||___|  |___||___|  |___||___| | |
| 1.17 | **What do you do of standing water collections around your house ?** | __________________________________________________________________________________________________________________ | | | |___||___|  |___||___|  |___||___| | |
| 1.18 | **Can you cite some symptomes of malaria ?** | __________________________________________________________________________________________________________________ | | | |___||___|  |___||___|  |___||___| | |
| 1.19 | **What do you do when you suspect a case of malaria?** | 1. Go to the hospital/clinic for consultation ; 2. Self medication ; 3. Traditional medicine   ***Order your choices 1, 2, 3*** | | | |___|  |___|  |___| | |
| 1.20 | **Where do you purchase your drugs ?** | 1= Pharmacy ; 2 = Hospital/Clinic ;  3 = Street sellers ; 4 = Traditional healers ;  5 = Other (give precision)__________________ | | | |___| | |
| 1.21 | **What amount do you spend annually ?** | 1. For fighting against mosquitoes 2. For the treatment of malaria | | | **in FCFA**  |___|___|___|___|  |___|___|___|___| | |
| 1.22 | **Will you approve mosquito collections in your house**? | 1= Yes 2= No | | | |___| | |

**Autres observations**__________________________________________________________________________

___________________________________________________________________________________________

**END OF THE INTERVIEW**.
